# *Syzygium aromaticum* essential oil and its major constituents: Assessment of activity against *Candida* spp. and toxicity

**DOI:** 10.1371/journal.pone.0305405

**Published:** 2024-06-18

**Authors:** Priscilla Guimarães Silva Vasconcelos, Gabriel Flores Abuna, Joanda Paolla Raimundo e Silva, Josean Fechine Tavares, Edja Maria Melo de Brito Costa, Ramiro Mendonça Murata

**Affiliations:** 1 Department of Dentistry, Postgraduate Program in Dentistry, State University of Paraiba, Campina Grande, Paraíba, Brazil; 2 Department of Foundational Sciences, School of Dental Medicine, East Carolina University, Greenville, North Carolina, United States of America; 3 Multi-User Laboratory for Characterization and Analysis, Federal University of Paraíba, João Pessoa, Paraíba, Brazil; Museu Paraense Emilio Goeldi, BRAZIL

## Abstract

*Syzigium aromaticum* essential oil (EO), eugenol, and β-caryophyllene were evaluated regarding antifungal, antibiofilm, and *in vitro* toxicity. Additionally, *in vivo* toxicity of EO was observed. Anti-*Candida* activity was assessed through broth microdilution assay for all compounds. Time-kill assay (0, 1, 10, 30 min, 1, 2, and 4 h) was used to determine the influence of EO and eugenol on *Candida* Growth kinetics. Thereafter, both compounds were evaluated regarding their capacity to act on a biofilm formation and on mature biofilm, based on CFU/ml/g of dry weight. Cell Titer Blue Viability Assay was used for *in vitro* cytotoxicity, using oral epithelial cells (TR146) and human monocytes (THP-1). Lastly, *Galleria mellonella* model defined the EO *in vivo* acute toxicity. All compounds, except β-cariofilene (MIC > 8000 μg/ml), presented antifungal activity against *Candida* strains (MIC 500–1000 μg/ml). The growth kinetics of *Candida* was affected by the EO (5xMIC 30 min onward; 10xMIC 10 min onward) and eugenol (5xMIC 10 min onward; 10xMIC 1 min onward). Fungal viability was also affected by 5xMIC and 10xMIC of both compounds during biofilm formation and upon mature biofilms. LD_50_ was defined for TR146 and THP1 cells at, respectively, 59.37 and 79.54 μg/ml for the EO and 55.35 and 84.16 μg/ml for eugenol. No sign of toxicity was seen *in vivo* up to 10mg/ml (20 x MIC) for the EO. *S*. *aromaticum* and eugenol presented antifungal and antibiofilm activity, with action on cell growth kinetics. *In vivo* acute toxicity showed a safe parameter for the EO up to 10 mg/ml.

## Introduction

Denture stomatitis is characterized as the inflammation of the oral mucosa underlying a removable denture. Thus, it commonly affects the palate and alveolar mucosa sites [1, 2]. The etiology of this condition is multifactorial and may be associated with prolonged use of dental prosthesis, local trauma, low salivary flow, hypersensitivity to the material that constitutes the prosthesis, and/or sub-optimal oral and denture hygiene [3–5]. Denture stomatitis is also considered the most prevalent clinical form of oral candidiasis, constituting 70–95% of the diagnosed cases, since local infection by *Candida* spp., especially *Candida albicans*, is frequently observed. The pathogen can find in the denture surface a protected environment to adhere and colonize, forming a biofilm that would be in direct contact to the oral mucosa, leading to tissue invasion and pathogenesis development [2, 6–8].

Adequate measures to solve most cases includes using antifungal agents [9]. However, available antifungal drugs are somewhat scarcer than antibacterial, and the rise of *Candida* resistance must not be underestimated. The noteworthy potential of natural products in combatting fungal infections has continually gained attention. Over the past four decades, 20% of the novel antifungal agents approved by the FDA have been derived from natural products, which underscores their potential in combatting fungal infections. However, despite the rising concern with fungal resistance, no antifungal derived from natural compounds has been registered since 2006, which increases the need for new research in this field [10].

Popularly known in Brazil as *cravo or cravo-da-índia* and as clove in English, *Syzygium aromaticum* is a medicinal plant that the population has vastly used to treat different disorders such as the treatment of burns and wounds, to treat infections in different sites, and as a pain reliever. Based on its widespread use, scientific works were performed to address its biological activities, and positive results have been attributed to this plant and its major constituents [11, 12]. *S*. *aromaticum* has gained attention due to its antimicrobial properties against gram-positive and gram-negative bacteria, and against yeast such as *Candida albicans* [13, 14]. Studies have shown that the permeability of phenolic substances such as eugenol to cell membranes and the irreversible disruption of cell membrane integrity are the main factors related to its antimicrobial action [12, 13, 15]. Antioxidant and anti-inflammatory properties have also been attributed to *S*. *aromaticum*. Thus, it can reduce free radical accumulation *in vivo*, decrease oxidative cellular damage, reduce the risk of diseases associated with oxidative stress [12, 16–18] and it can regulate inflammatory cascades via reducing pro-inflammatory biomarkers and cytokines such as IL-1β and IL-6 [12, 18]. Additional properties have also been reported in the literature, such as neuroprotective [18, 19], anticancer [12, 20], antinociceptive and analgesic activity [12, 21]. The biological activity of *S*. *aromaticum* may be attributed to its chemical composition, and among the major constituents of the oil we can highlight eugenol and β-Caryophyllene [14, 15, 22].

Based on *S*. *aromaticum* biological potential our group conducted a preliminary study and a good potential of *S*. *aromaticum* essential oil against a multispecies biofilm derived from patients with oral candidiasis was seen [14]. However, aiming to focus its application as a mouthwash that acts controlling *Candida* virulence factors in denture stomatitis, we need to approximate the test conditions to the clinical conditions in which it would be used. Thus, one-minute treatments needed to be employed instead of the usual 24 hours used at *in vitro* tests. *In vitro* and *in vivo* toxic potential were also analyzed since those are essential steps that would guide future clinical studies with the compound. Therefore, the present study aims to evaluate the capacity of *S*. *aromaticum* essential oil and its major compounds, eugenol and β-caryophyllene, to act on *C*. *albicans* viability, growth kinetics, and biofilm formation, as well as to establish the essential oil toxicity *in vitro* and *in vivo*.

## Materials and methods

### Essential oil

The present study used *S*. *aromaticum* flower bud’s essential oil (Laszlo Aromaterapia Eireli, BH—Brazil), eugenol (Spectrum Chemical MFG, NJ–United States), and β-cariofileno (Pfaltz & Bauer, CT–United States).

### Phytochemical evaluation

The sample was analyzed by gas chromatograph (GC) coupled to mass spectrometer (MS) with electron impact ionization (EI) at 70 eV (model GCMS-QP2010 Ultra, Shimadzu), with RTX-5MS chromatographic column (30 m/0.25 mm/0.25 μm). The temperature of the injector was 220°C, and the initial temperature was 60°C with a temperature gradient of 240°C in the ratio of 3°C/minute. The gas flow (Helium) was 1.1 ml/minute, and the injection mode was performed by split. Mass spectra were scanned from *m/z* 30–600. The data were compared with commercial Wiley08 and NIST08 libraries, and retention indices (RI) of metabolites were calculated using n-alkanes (C7-C40, Sigma-Aldrich) analyzed in the exact parameters of GC-MS from the sample [14].

### Evaluation of antifungal activity

#### Microorganisms

The following standard ATCC (American Type Culture Collection) reference yeast of *Candida* were used: *C*. *albicans* ATCC 321182, *C*. *albicans* ATCC *90028*, *C*. *albicans* ATCC MYA 2876, *C*. *albicans* ATCC MYA 274, *C*. *tropicalis* MYA 750, *C*. *dubliniensis* ATCC MYA 646, and *C*. *glabrata* ATCC MYA 275.

#### Determination of Minimal Inhibitory Concentration (MIC) and Minimal Fungicidal Concentration (MFC)

The microdilution method was used [23] to determine the MIC and MFC of the *Candida* strains. Roswell Park Memorial Institute Medium—RPMI-1640 (Corning^®^) was inserted into the wells, followed by different concentrations of *S*. *aromaticum* essential oil (2000 to 15.6 μg/ml), eugenol (2000 to 15.6 μg/ml), β-Caryophyllene (8000 to 62.5 μg/ml), and fluconazole (Sigma-Aldrich^®^) (256 to 0.12 μg/ml), diluted in 1% of dimethyl sulfoxide (DMSO). Lastly, fungal suspension (2.5 × 10^3^ colony forming units - CFU/ml) were added to the wells. Wells containing DMSO 1%, inoculum and medium were used as the vehicle control. Plates were incubated at 37°C– 5% CO_2_ for 24 h, and microbial growth was observed visually. Later, 10 μl of each well, with equal and/or higher concentrations of MIC were sub-cultured in sabouraud dextrose agar (Kasvi^®^) at 37°C– 5% CO_2_ for 24 h, and the visual growth was analyzed to determine the MFC. The ratio between MFC and MIC was used to determine the compound’s behavior as fungicidal (MFC/MIC<4) or fungistatic (MFC/MIC ≥ 4).

#### Time-kill assay

Based on MIC and MFC results, *S*. *aromaticum* essential oil and eugenol were evaluated on the growth kinetics of *C*. *albicans* ATCC MYA 2876. *S*. *aromaticum* essential oil were used at 2500 and 5000 μg/ml and eugenol at 5000 and 10000 μg/ml, respectively equivalent to 5xMIC and 10xMIC. The following controls were also added to the test: fluconazole 10 μg/ml (10xMIC) as the positive control, DMSO 1% and medium as a negative control. Testing samples were added as 10% of the inoculum final volume, which was defined as 10^6^ CFU/ml. The solution was placed on a shaker and incubated at 37°C– 5% CO_2_. Thereafter, samples of 10 μl were plated on sabouraud dextrose agar at predetermined time points (0, 1, 10, 30 min, 1, 2, and 4 h) and after 48 h visual growth was analyzed to establish CFU/ml number [14, 24].

#### Determination of antibiofilm potential

At this stage, we used one-minute treatment, simulating a mouthwash swish, to evaluate *S*. *aromaticum* essential oil and eugenol capacity to inhibit biofilm formation and to act against a mature biofilm under this condition.

To evaluate the biofilm formation inhibition, an inoculum of 1x10^6^ CFU/ml of *C*. *albicans* ATCC MYA-2876 was prepared using Yeast Nitrogen Base Medium (YNB) (Sigma Aldrich, Saint Luis, MO) supplemented with 50 mM of glucose (VWR Life Science, Radnor, PA) for 24 h at 37°C—5% CO_2_ to establish initial biofilm growth. After 24 hours of incubation, the biofilm was treated daily, until it completed 72 hours, with 10% v/v of the samples prepared in 1% DMSO as the vehicle. *S*. *aromaticum* essential oil was used at 500, 2500, and 5000 μg/ml and eugenol at 1000, 5000, and 10000 μg/ml, concentrations respectively equivalent to MIC, 5xMIC, and 10xMIC. At each 24 hours’ time, the supernatant was removed, and samples were added for one-minute treatments afterwards treatments were removed, the biofilm was washed twice with Phosphate Buffer Solution (PBS) (Lonza Bioscience, Walkersville, MD), and 1 ml of fresh YNB medium was added to the wells. The plates were incubated at 37°C—5% CO_2_ for 24 h, and this process was repeated until 72 hours of treatment were completed. The vehicle control was 1% DMSO, while the positive control was Fluconazole 10 μg/ml (10xMIC).

The mature biofilm was formed following the same concept described above. However, the biofilm remained untouched for 72 h. Treatments were also applied as described. After the treatment time of both methods, adhered biofilms were collected by scraping the bottom of each well plate and suspending in PBS, which was then centrifuged at 10,000 rpm for 5 minutes. The biomass (dry weight) of each biofilm sample was obtained by discarding the supernatant and placing the samples in a speed vacuum to dry for 40 minutes. CFU was determined by counting the colonies at Sabouraud Dextrose Agar plates, which were incubated at 37°C—5% CO_2_. Data was normalized based on the CFU/ml/dry weight of biofilm sample [24–27].

### Cytotoxicity assay

#### Cytotoxicity assay with human squamous cell carcinoma (TR146) and human monocytes (THP-1)

The *in vitro* cytotoxic effect of *S*. *aromaticum* essential oil and eugenol was performed with concentrations ranging from 2500 to 0.25 μg/ml. The resazurin fluorometric method (Cell Titer Blue Viability Assay, Promega Corp^®^, WI—United States) was employed using both THP-1 (ATCC TIB-202) and TR146 (ECACC 10032305) cells. DMSO with a final concentration in the wells of 0.1% was used as the vehicle.

THP-1 cells were cultured in RPMI medium (FBS Gibco, Invitrogen, MA—United States) and kept at 37°C—5% CO_2_ for 48–96 h. Thereafter, an inoculum of 2.5x10^5^ cells/ml was seeded in a 24-well plate in fresh medium, followed by the compound’s addition (10% v/v) in the predetermined concentrations. On the other hand, TR146 cells were cultured in Ham`s F12 medium with L-glutamine (Lonza Bioscience^®^, MD—United States), supplemented with 10% of FBS and Penicillin/Streptomcin. Cells (1x10^6^ cells/ml) were initially seeded with fresh medium only in a 24-well plate until it reached confluency. Medium changes were made every 2–3 days. Then, cells were washed with PBS, and the treatment was added (10% v/v) as mentioned above. The plates were incubated for 24 h at 37°C—5% CO_2_.

Afterwards, for both experiments, cell titer blue was added to each well, following a proportion of 20 μL of the reagent to each 100 μL of medium. Cells were then incubated for 3 h. The fluorescence of the supernatant was read in a microplate reader with excitation of 555 nm and emission of 585 and 570 nm cut off [28].

#### *In vivo* toxicity of geraniol in *G*. *mellonella* larvae model

Different doses of *S*. *aromaticum* essential oil were tested following an increasing order up until 10 mg/ml (MIC, 2xMIC, 5xMIC, 10xMIC, 15xMIC, and 20xMIC) to obtain the *in vivo* acute toxicity in a *G*. *mellonella* model. A random selection of 10 healthy-looking larvae weighing between 0.2 and 0.3 g was made for each group. A volume of 5 μL of each treatment and control were injected into the left proleg of the larvae using a 25 μL Hamilton Syringe (Hamilton, Reno, NV). Three controls were added to the test: 1) treatment control, larvae subjected to the injection only; 2) vehicle control—1% DMSO; 3) toxicity control—DMSO 100%. The larvae were incubated at 30°C and their survival was evaluated until the maximum of 96 h. The pathological scoring system described by Loh et al [29] and Champion et al [30] was used to allow subtle differences in larval health to be assessed based on their appearance; the following parameters were analyzed: larvae activity, cocoon formation, degree of myelinization, and larvae survival. Altogether, toxicity was considered by comparing the treatment group with the controls. Based on the parameters expressed on Table 1 a healthy larvae would score between 9 and 10 [31].

**Table 1:** The *G*. *mellonella* health index scoring system [29, 30].

Category	Description	Score
Activity	No movement	0
	Minimal movement on stimulation	1
	Move when stimulated	2
	Move without stimulation	3
Cocoon formation	No cocoon	0
	Partial cocoon	0.5
	Full cocoon	1
Melanisation	Black larvae	0
	Black spots on brown larvae	1
	≥3 spots on beige larvae	2
	< 3 spots on beige larvae	3
	No myelinization	4
Survival	Dead	0
	Alive	2

### Statistical analysis

All *in vitro* analysis were realized in triplicates in three distinct times. When applicable, the results were expressed as mean and standard deviation, and the raw data obtained in the present study can be found in the S1 File. Data were statistically analyzed using GraphPad Prism software (version 8.02). Differences between groups were analyzed using parametric or non-parametric measures, as dictated by the results. In the time-kill assay, Friedman, followed by the Kruskal-Wallis test, were used. One-way analysis of variance (ANOVA) and Dunnett’s multiple comparison tests in relation to the negative or vehicle control were applied in biofilm and cytotoxic analysis. Lastly, non-linear regression assessed LD_50_ for cytotoxic, and the Kaplan-meier survival analysis was applied in the *G*. *mellonella in vivo* analysis. Significance was accepted for a value of p≤ 0.05.

## Results

### Phytochemical evaluation

Eugenol (82.71%) and β-Cariofillene (9.06%) were the major constituents identified in *S*. *aromaticum* essential oil; other constituents are described in Table 2.

**Table 2:** Chemical composition of *S*. *aromaticum* essential oil identified by GC-MS.

COMPOUND	RT	IR	(%)
Eugenol	22.21	1282	82.71
β-Caryophyllene	24.69	1411	9.06
α-Humulene	26.13	1429	0.74
Eugenol acetate	29.24	1466	7.49

RT = Retention time; IR = Retention index; (%) = Fraction in percentage of the total integrated area for the chromatogram.

### Evaluation of antimicrobial activity

#### Determination of Minimal Inhibitory Concentration (MIC) and Minimal Fungicidal Concentration (MFC)

*S*. *aromaticum* essential oil and eugenol presented antifungal activity against *albicans* and non-*albicans Candida* strains (MIC 500–1000 μg/ml, MFC 1000–2000 μg/ml), however, such effect was not observed with β-Cariofillene (MIC and MFC > 8000 μg/ml). Values of MIC and MFC, as well as MFC/MIC ratio [32], for all *Candida* strains tested are presented in Table 3.

**Table 3:** Minimal Inhibitory Concentration (MIC) and Minimal Fungicidal Concentration (MFC) for *S*. *aromaticum* essential oil, eugenol, ß-caryophyllene, and fluconazole according to *Candida* species.

Microorganisms	*S*. *aromaticum* essential oil			Eugenol			ß-cariofillene			Fluconazole		
	MIC μg/ml	MFC μg/ml	MIC/MFC	MIC μg/ml	MFC μg/ml	MIC/MFC	MIC μg/ml	MFC μg/ml	MIC/MFC	MIC μg/ml	MFC μg/ml	MIC/MFC
*C*. *albicans* ATCC 321182	500	2000	4	500	2000	4	>8000	>8000	-	64	256	4
*C*. *albicans* ATCC MYA 274	1000	2000	2	1000	1000	1	>8000	>8000	-	0.12	32–64	>4
*C*. *albicans* ATCC MYA 2876	500	2000	4	1000	2000	2	>8000	>8000	-	1	128	>4
*C*. *albicans* ATCC MYA 90028	500	2000	4	1000	2000	2	>8000	>8000	-	0.25	64	>4
*C*. *dublinienses* ATCC MYA 646	500	2000	4	1000	1000	1	>8000	>8000	-	0.12	128	>4
*C*. *tropicalis* ATCC 750	1000	2000	2	1000	1000	1	>8000	>8000	-	1	>256	>4
*C*. *glabrata* ATCC MYA 275	250	1000	4	1000	2000	2	>8000	>8000	-	0.5	256	>4

Fungicidal (MFC/MIC<4) and fungistatic (MFC/MIC ≥ 4) [32].

### Time-kill assay

**Fig 1:**
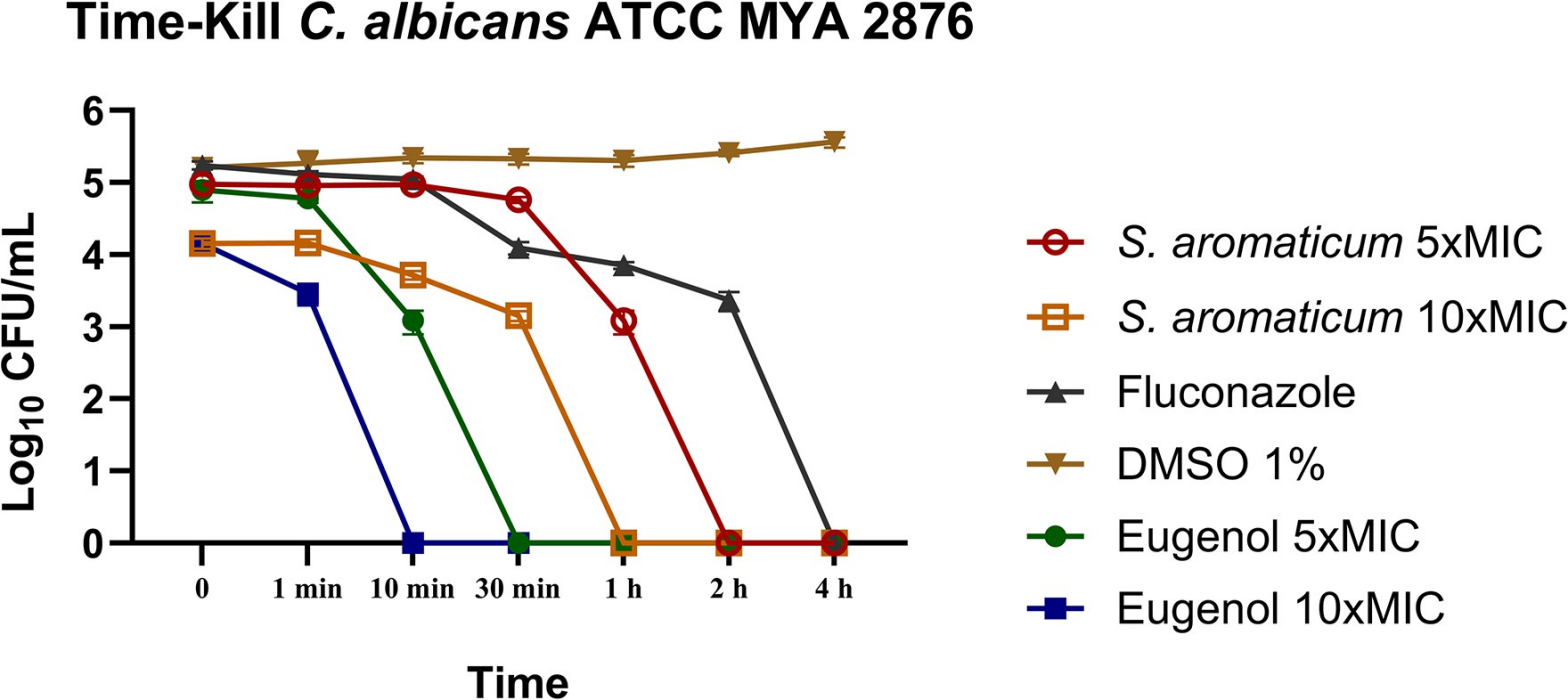
*S*. *aromaticum* essential oil (5xMIC—2500 μg/ml and 10xMIC 5000 μg/ml) and eugenol (5xMIC– 5000 μg/ml and 10xMIC—10000 μg/ml) action upon *C*. *albicans* ATCC MYA 2876 growth kinetics. DMSO 1%: Vehicle control; Fluconazole (10xMIC– 10 μg/ml): positive control.

*S*. *aromaticum* essential oil 5xMIC and 10xMIC, were able to interfere significantly in *C*. *albicans* ATCC MYA 2876 growth kinetics, respectively, from 30 and 10 min onward when compared to the vehicle control (DMSO 1%). The points at which no visible growth was seen in the plates were 2h and 1h respectively. Regarding eugenol treatment, a significant difference in growth kinetics was seen for 5xMIC and 10xMIC, respectively, from 10 and 1 min onward. No visual growth was seen, respectively, from 30 and 10 min onward (Fig 1).

### Determination of antibiofilm potential

**Fig 2:**
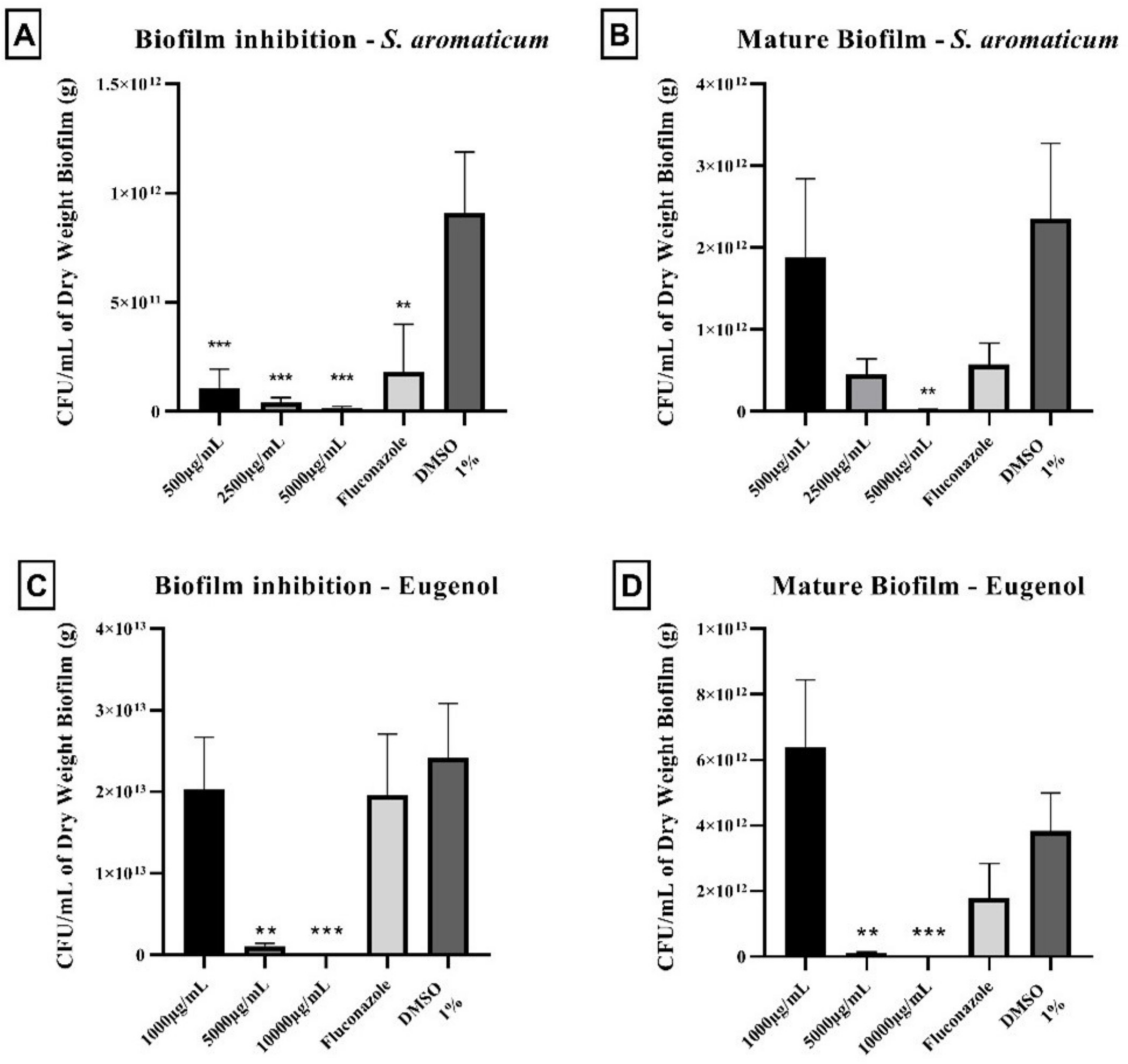
*S*. *aromaticum* essential oil (A and B) and eugenol (C and D) action upon biofilm inhibition and against a mature biofilm of *C*. *albicans*. After the 1 min/day treatment both compounds were able to reduce fungal viability during biofilm formation, as well as, 5xMIC and 10xMIC could statistically reduce the yeast load of a mature biofilm (*p < 0.05; **p<0.001; ***p<0.0001; significance values were compared to vehicle control).

All tested concentrations of *S*. *aromaticum* essential oil and eugenol were capable of statistically (p<0.05) reduce fungal viability during biofilm formation after the 1 min/day treatment. Regarding mature biofilms, only the concentrations equivalents to 5xMIC and 10xMIC could statistically (p<0.05) reduce the yeast load in comparison to the vehicle control group (DMSO 1%) (Fig 2).

### Cytotoxicity assay

**Fig 3:**
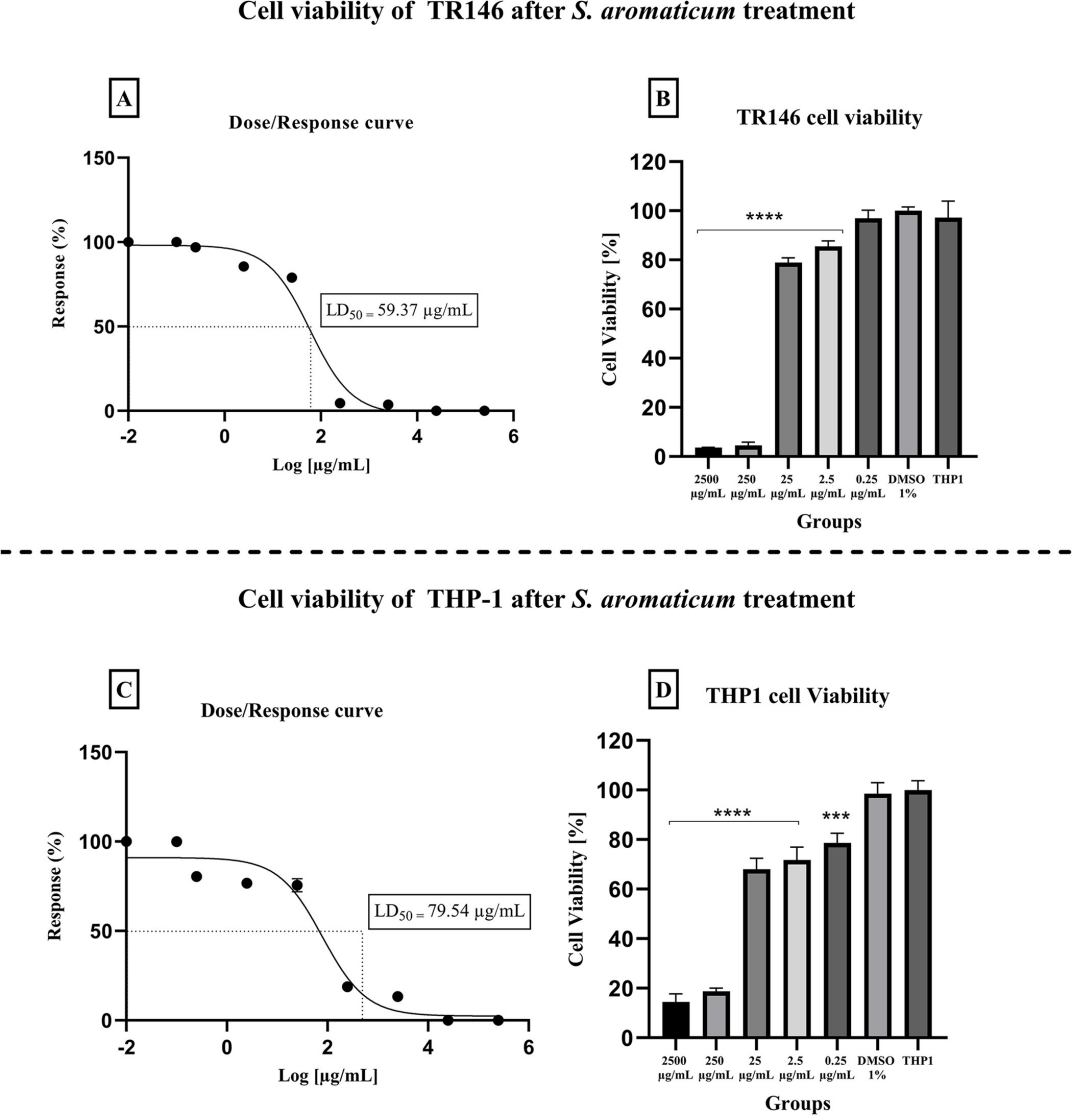
Cytotoxic effect of *S*. *aromaticum* essential oil (0.25–2500 μg/ml) on TR146 and THP-1 cells after 24 hours of treatment. LD_50_ for TR146 (A and B) and THP-1 (C and D) cells were 59.37 and 79.54 μg/ml. TR146 and THP-1: Cells only; DMSO 0.1%: Vehicle control. (*p ≤ 0.05, **p ≤ 0.01, ***p ≤ 0.001, and ****p ≤ 0.0001; significance values were compared to vehicle control).

**Fig 4:**
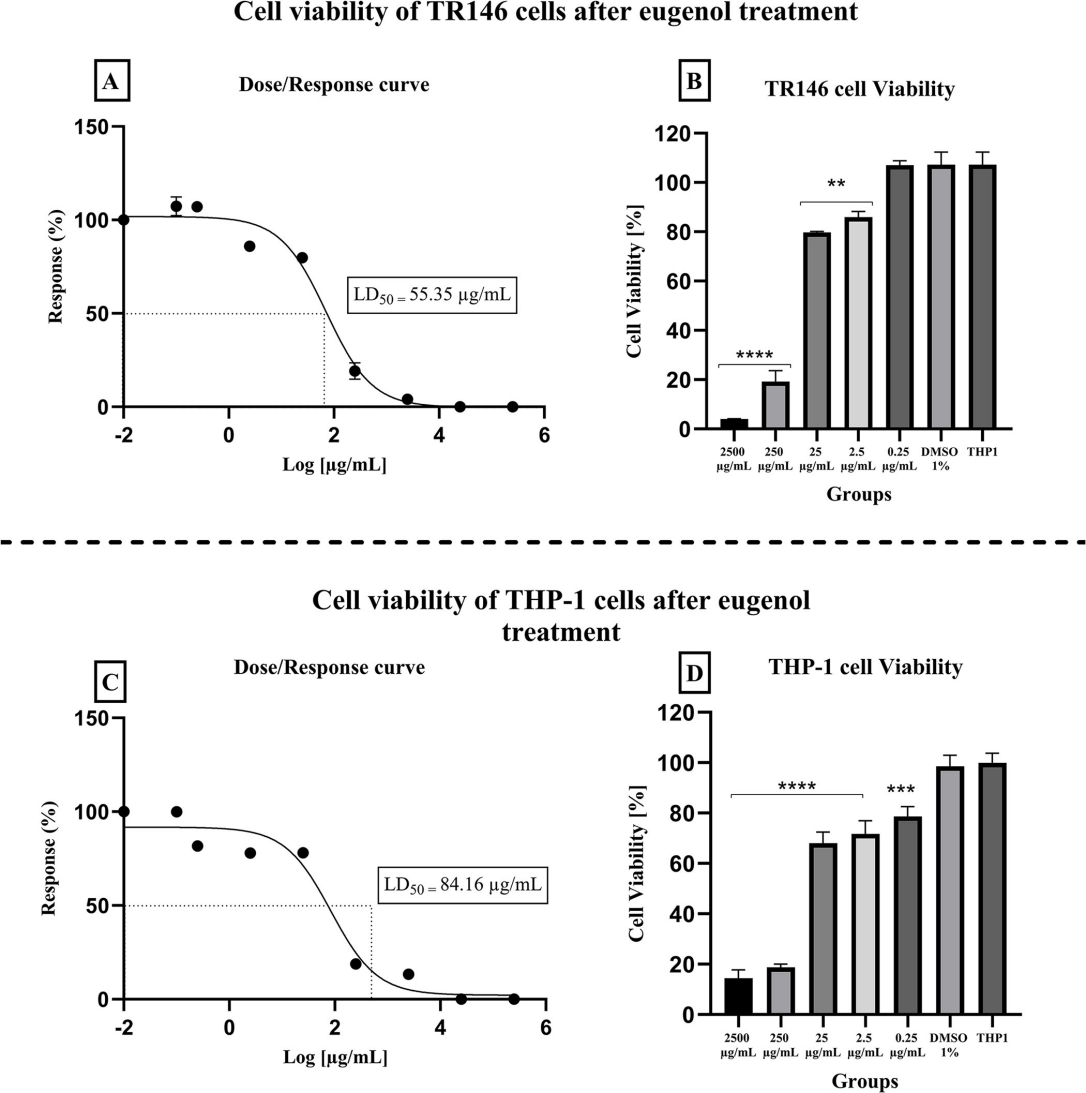
Cytotoxic effect of eugenol (0.25–2500 μg/ml) on TR146 and THP-1 cells after 24 hours of treatment. LD_50_ for TR146 (A and B) and THP-1 (C and D) cells were 55.35 μg/ml for TR146 and 84.16 μg/ml. TR146 and THP-1: Cells only; DMSO 0.1%: Vehicle control. (*p ≤ 0.05, **p ≤ 0.01, ***p ≤ 0.001, and ****p ≤ 0.0001; significance values were compared to vehicle control).

The LD50s of *S*. *aromaticum* essential oil for TR146 and THP-1 cells were 59.37 and 79.54 μg/ml (Fig 3), respectively. The LD50 of eugenol was established at 55.35 μg/ml for TR146 and 84.16 μg/ml for THP-1 cells (Fig 4).

### *In vivo* toxicity of geraniol in *G*. *mellonella* larvae model

No sign of toxicity was seen in the larvae under *S*. *aromaticum* treatment up to 10 mg/ml (20 x MIC), whereas in the toxicity control (DMSO 100%) all larvae died within the first 2 hours of treatment. The signs of toxicity were assessed compared to the vehicle control (DMSO 1%). The obtained scores are available in the S1F Table of the S1 File.

## Discussion

The protocol of one-minute treatment with *S*. *aromaticum* essential was able to reduce *C*. *albicans* viability during biofilm formation as well as to decrease CFU/ml/g of dry weight in the mature biofilm. This result is relevant to support the possible use of *S*. *aromaticum* in a mouthwash formulation with antifungal properties, guiding its appliance toward denture stomatitis treatment.

A previous study evaluating *S*. *aromaticum* antifungal activity was conducted by our research group, and the results showed that the *S*. *aromaticum* essential oil could inhibit the formation of a multispecies biofilm derived from the saliva of patients diagnosed with oral candidiasis, in which *C*. *albicans* and non-*albicans Candida* were presumptively identified alongside with different bacteria species [14]. However, in that study, as well as in others, 24 h treatment was used, which would not match a mouthwash treatment [14, 37, 38].

Based on Holetz et al [33], our results showed a moderate antifungal activity (100μg/ml < MIC ≤ 500 μg/ml) against all *Candida* tested, except for *C*. *glabrata* upon which the essential oil presented a strong activity (MIC < 500μg/ml). In addition to the non-albicans *Candida* evaluated in the present study, positive results have been documented for *C*. *krusei* and *C*. *parapisilosis* [34], which constitute a positive remark since the relation with oral candidiasis, as well as to antifungal resistance of non-albicans *Candida*, such as *C*. *glabrata*, is a relevant point of discussion in recent studies [35], thus, the strong activity showed for *C*. *glabrata* strain must be taken in consideration and further studies could be conducted to better investigate the possible *S*. *aromaticum* effect upon non-albicans *Candida* virulence factors.

Similar MIC values to the ones found in the present study were documented by other studies such as Hekmatpanah et al [15] (MIC 625–1250 μg/ml) and Vasconcelos et al [14] (MIC 500–1000 μg/ml), whereas, a lower value of 100 μg/ml was obtained from Condó et al [36] for *C*. *albicans* 10231, these difference may be related to the different strain used or differences in the methodology. Furthermore, a fungistatic pattern was seen for most of the tested yeast in the present study. The fungistatic profile of a compound might constitute a desirable effect rather than the complete elimination of the pathogen [37, 38]. *Candida* spp. is an essential component of the oral microbiome, present in immunocompetent individuals as a commensal pathogen. Thus, controlling its virulence factors should prevent the rise of pathogenic strains and maintain microbiome homeostasis [38, 39].

Phytochemical analysis obtained in the present study, alongside previous studies, has shown that eugenol and β-caryophyllene are major compounds of *S*. *aromaticum* essential oil. Thus, it might be related to the main biological activities of the plant [14, 15, 22]. However, regarding the antifungal properties, our results showed that only eugenol was effective, and its MIC range was the same as the essential oil, 500–1000 μg/ml, however the prevalence of MICs at 1000 μg/ml was slightly higher. Thus, we could infer that the conjunct action of the compounds presented in the phytocomplex that is the essential oil could have enhanced the antifungal activity, resulting in a slight difference in MIC ranges between the essential oil and the isolated compound. Other authors have compared MICs of *S*. *aromaticum* essential oil and eugenol and similar values were also found, enhancing the possibility that antimicrobial properties are associated with eugenol [22, 34, 40]. For instance, Biernasiuk et al [22] compared the antifungal activity of *S*. *aromaticum* and eugenol against 5 reference strains and 60 clinical oral isolates of *C*. *albicans*. The authors found a similar activity for both compounds at MICs in 0.25–2 mg/ml range, in which most *Candida* isolates were inhibited at a minimal concentration of 0.5 mg/ml, similar to the one found in the present study.

Furthermore, *S*. *aromaticum* essential oil and eugenol appear to have similar mode of action upon interaction with *Candida* strains, underscoring the correlation of eugenol in *S*. *aromaticum* essential oil biological activity. Given that, Biernasiuk et al [22] suggested that both compounds appear to bind to the ergosterol in the membrane, which increases ion permeability and ultimately results in cell death. The impact on membrane proteins and secreted enzymes, such as proteinases, which are important for hyphal development, has also been documented for both compounds, as well as the reduction of *C*. *albicans* germ tubes formation [41–43]. Knowing the components responsible for the essential oil’s major biological activities is an important step towards getting to know its mode of action. However, the synergistic interaction between two or more components in the phytocomplex must influence anti-virulence activity and, therefore, influence its outcome.

Although no antifungal activity was seen for β-caryophyllene, other properties have been addressed in the literature, such as the analgesic and antiiflammatory activity [44], as well as wound healing capability [45]. In view of a treatment for oral candidiasis, a condition that can be approached as multifactorial with a fungal and inflammatory constituent [39], those properties may be a promising addition to the formulated compound since it could act on the dysregulated inflammatory axis induced by *Candida* spp. Additionally, some papers have shown the antiinflammatory activity of *S*. *aromaticum* essential oil. However, further studies could be conducted to underscore the antiinflammatory role of this compound and its relation to its major constituents upon *Candida* infection. A bioactive compound that could act both in modulating the virulence factors of *C*. *albicans* and on host’s inflammatory response against the pathogen would likely improve the clinical response to the treatment.

The time-kill analysis performed in a previous study conducted by our research group [14] showed that concentrations of 2000 μg/mL and 1000 μg/mL were able to respectively reduce *Candida* growth from 2h and 4h onward, time points started at 1h. However, to simulate a mouthwash use, the time of contact would need to be reduced, and allegedly, concentration would need to be higher. Thus, 5xMIC and 10xMIC were used here. Additionally, most published studies only used time points equal to or higher than 1 h [46, 47]. For instance, Fazly et al [46] observed the reduction of *Candida* growth kinetics at a starting point of 3 h after *S*. *aromaticum* treatment. Therefore, time points of 1, 10 and 30 min were added to the test.

Our results showed that the essential oil at 10xMIC were able to promote a significant reduction in the number of CFU/ml within the first 10 minutes of contact with the pathogen, whereas eugenol 10xMIC had the same effect within the first minute of contact. The period of inhibition of cell growth in the graphs shows that the strains failed to reach the Log phase, a phase of great cellular enzymatic activity [47, 48], underscoring the possibility of *S*. *aromaticum* action in the enzymatic activity inhibition and the increase in cell permeability [22, 41–43, 49]. However, further studies should be conducted to evaluate its action upon *Candida* virulence factors to establish the essential oil effect on *Candida* pathogenesis.

Assessing the compound’s *in vitro* and *in vivo* toxic parameters is an important step to future clinical studies. In our findings, both *S*. *aromaticum* and eugenol had similar LD_50_ values for each tested cell type. LD_50_ obtained for TR146 cells were 59.37 and 55.35 μg/ml, whereas for THP-1 was 79.54 and 84.16 μg/ml, respectively, for *S*. *aromaticum* essential oil and eugenol. Other studies have also analyzed *S*. *aromaticum* cytotoxicity; for instance, in Ribeiro et al [50], *S*. *aromaticum* essential oil only showed cytotoxic for keratinocytes at the highest concentration of 250 μg/ml, similar to our study, in which the 250 μg/mL reduced the cell viability in over 70%. Regarding eugenol, Ranjitkar et al [51] evaluated cytotoxic parameters using fibroblasts and noticed that eugenol showed a dose-dependent cytotoxic effect. Exerting significant effect on cell viability only at concentrations higher than 400 μg/mL, a higher concentration compared to the one obtained in the present study.

The concentration obtained in the *in vitro* cytotoxicity test for both compounds was considered low when compared to MIC (500–1000 μg/ml) values, which would mean that using the compound in the MIC concentration would probably cause a distress in the cells. Moreover, we intended to use even higher doses (5xMIC and 10xMIC) in the biofilm test due to the stable environment formed by a structured biofilm associated to the reduced treatment time (one minute). However, *in vitro* tests with cell lines are considered sensible, in which a component can appear toxic by being applied in direct contact with the cell lines, whereas further *in vivo* tests that use more complex organisms may show safety parameters.

Following cytotoxicity tests, *S*. *aromaticum* was tested in the *G*. *mellonella in vivo* model to evaluate its acute toxicity. The innate immune response of *G*. *mellonella* shares several properties with the mammalian immune system, and it qualifies as a well-accepted scientific method to be used as a preclinical stage [52]. Our findings show that the *S*. *aromaticum* essential oil presented no toxic effect on the larvae up to 10 mg/ml (20xMIC). Further tests could include infecting the larvae with *C*. *albicans* and analyzing the effect of *S*. *aromaticum* essential oil treatment upon infection; it would be an additional parameter to subside future clinical studies. To the best of our knowledge, there is no other studies evaluating *in vivo* toxicity of *S*. *aromaticum* with *G*. *mellonella* model; however, *in vivo*, toxicity of this compound was evaluated in mice, and a value of 4500 mg/kg was found [53]. Furthermore, the US Food and Drug Administration (FDA) has approved clove buds, clove oil, and oleoresins as generally recognized as safe (GRAS) [54] however, although we have a sound indicative of the essential oil safety, the obtained results should be used to sustain future *in vivo* studies to affirm its safe use and attest efficacy in oral candidiasis treatment.

The properties of *S*. *aromaticum* essential oil have underscored its use in different delivery systems reported by previously published studies, which in the majority used the essential oil in nanotechnology-based delivery systems. The nanoemulsions showed positive results regarding antiinflammatory, antimicrobial, and anticancer activities [20, 55, 56]. However, despite the promising antifungal activity, few studies aimed to develop a compound to be used as an antifungal agent. Thus, two studies can be highlighted. Shehabeldine et al. [20] developed an *S*. *aromaticum* nanoemulsion with promising antifungal activity against different fungus strains, including *C*. *albicans*. Whereas Jayasankar et al [57] combined *S*. *aromaticum* essential oil and *Origanum vulgare* in an herbal gel, obtaining a synergistic antifungal activity against oral *C*. *albicans*.

Our findings elucidate the safety *in vivo* parameters of using *S*. *aromaticum* essential oil up until 20xMIC, as well as its antibiofilm capacity under one-minute treatments. The test conditions used in the present study approximated the clinical conditions of which a mouthwash would be used, underscoring the possible use of this formulation as an alternative or complementary therapy in oral candidiasis treatment since no clinical studies with this configuration have been published yet. However, further investigations may be done regarding the essential oil action upon other *C*. *albicans* key virulence factors, such as cell surface adhesins expression, proteolytic enzyme activity, host immune factors degradation, and host tissue invasion and destruction mechanisms. Lastly, more robust *in vivo* and clinical tests should follow to assure its efficacy in the treatment and/or the prevention of oral candidiasis.

## Conclusions

The present study showed *S*. *aromaticum* and eugenol antifungal activity against *C*. *albicans* and non-*albicans Candida* species and both compounds reduced cell growth kinetics of *C*. *albicans*. Antibiofilm capacity was assessed under one-minute treatment and both compounds were able to inhibit biofilm formation and reduce viability in a mature biofilm. Finally, *in vivo* analysis using *G*. *mellonella* model showed a safe parameter for *S*. *aromaticum* essential oil up until 10mg/ml (20xMIC).

## Supporting information

S1 File Raw data. Values behind the means, other measures reported, and values used to build graphs.
(DOCX)
