# Supplementary material for: Syzygium aromaticum essential oil and its major constituents: Assessment of activity against Candida spp. and toxicity
Source: PLoS One. 2024 Jun 18;19(6):e0305405. doi: 10.1371/journal.pone.0305405 (PMC11185461; doi:10.1371/journal.pone.0305405)
Supplement: S1 File — Values behind the means, other measures reported, and values used to build graphs. (DOCX) [file pone.0305405.s001.docx]

**Supporting information**

**S1 Table A:** Raw data of *S. aromaticum* essential oil (5xMIC - 2500 µg/ml and 10xMIC 5000 µg/ml) and eugenol (5xMIC – 5000 µg/ml and 10xMIC - 10000 µg/ml) action upon *C. albicans* ATCC MYA 2876 growth kinetics. Results are expressed as CFU/ml number.

| **Samples** | **Time points** | | | | | |  |
| --- | --- | --- | --- | --- | --- | --- | --- |
|  | 0 | 1 min | 10 min | 30 min | 1 h | 2 h | 4h |
| *S. aromaticum* 5xMIC | 84000 | 85000 | 96000 | 54000 | 1000 | 0 | 0 |
|  | 86000 | 96000 | 95000 | 56000 | 1000 | 0 | 0 |
|  | 74000 | 78000 | 88000 | 64000 | 1000 | 0 | 0 |
|  | 110000 | 91000 | 82000 | 66000 | 1000 | 0 | 0 |
|  | 121000 | 98000 | 87000 | 51000 | 2000 | 0 | 0 |
|  | 108000 | 89000 | 99000 | 53000 | 1000 | 0 | 0 |
|  | 86000 | 97000 | 98000 | 56000 | 1000 | 0 | 0 |
|  | 84000 | 88000 | 101000 | 64000 | 1000 | 0 | 0 |
|  | 94000 | 99000 | 93000 | 54000 | 2000 | 0 | 0 |
| *S. aromaticum* 10xMIC | 10000 | 18000 | 5000 | 1100 | 0 | 0 | 0 |
|  | 14000 | 12000 | 5100 | 1300 | 0 | 0 | 0 |
|  | 16000 | 15000 | 6100 | 1800 | 0 | 0 | 0 |
|  | 16000 | 14000 | 4900 | 1500 | 0 | 0 | 0 |
|  | 18000 | 14000 | 6100 | 1500 | 0 | 0 | 0 |
|  | 14000 | 14000 | 4100 | 1500 | 0 | 0 | 0 |
|  | 14000 | 13000 | 4300 | 1200 | 0 | 0 | 0 |
|  | 13000 | 16000 | 5500 | 1300 | 0 | 0 | 0 |
|  | 14000 | 15000 | 5800 | 1600 | 0 | 0 | 0 |
| Eugenol 5xMIC | 84000 | 74000 | 1000 | 0 | 0 | 0 | 0 |
|  | 86000 | 66000 | 1000 | 0 | 0 | 0 | 0 |
|  | 74000 | 64000 | 1000 | 0 | 0 | 0 | 0 |
|  | 91000 | 51000 | 1000 | 0 | 0 | 0 | 0 |
|  | 12000 | 54000 | 2000 | 0 | 0 | 0 | 0 |
|  | 98000 | 65000 | 1000 | 0 | 0 | 0 | 0 |
|  | 86000 | 56000 | 1000 | 0 | 0 | 0 | 0 |
|  | 84000 | 49000 | 1000 | 0 | 0 | 0 | 0 |
|  | 94000 | 64000 | 2000 | 0 | 0 | 0 | 0 |
| Eugenol 10xMIC | 10000 | 2000 | 0 | 0 | 0 | 0 | 0 |
|  | 14000 | 4000 | 0 | 0 | 0 | 0 | 0 |
|  | 16000 | 4000 | 0 | 0 | 0 | 0 | 0 |
|  | 16000 | 2000 | 0 | 0 | 0 | 0 | 0 |
|  | 18000 | 3000 | 0 | 0 | 0 | 0 | 0 |
|  | 14000 | 2000 | 0 | 0 | 0 | 0 | 0 |
|  | 14000 | 4000 | 0 | 0 | 0 | 0 | 0 |
|  | 13000 | 3000 | 0 | 0 | 0 | 0 | 0 |
|  | 14000 | 2000 | 0 | 0 | 0 | 0 | 0 |
| Fluconazole | 180000 | 120000 | 100000 | 11000 | 8000 | 2000 | 0 |
|  | 160000 | 130000 | 110000 | 11000 | 7000 | 3000 | 0 |
|  | 190000 | 130000 | 120000 | 16000 | 7000 | 2000 | 0 |
|  | 180000 | 130000 | 120000 | 10000 | 6000 | 2000 | 0 |
|  | 180000 | 140000 | 100000 | 16000 | 8000 | 3000 | 0 |
|  | 190000 | 150000 | 110000 | 11000 | 8000 | 3000 | 0 |
|  | 190000 | 130000 | 120000 | 15000 | 7000 | 2000 | 0 |
|  | 120000 | 140000 | 120000 | 12000 | 7000 | 1000 | 0 |
|  | 170000 | 100000 | 100000 | 10000 | 6000 | 3000 | 0 |
| DMSO 1% | 170000 | 80000 | 160000 | 170000 | 160000 | 250000 | 330000 |
|  | 100000 | 10000 | 180000 | 200000 | 180000 | 260000 | 490000 |
|  | 180000 | 180000 | 220000 | 180000 | 210000 | 220000 | 410000 |
|  | 180000 | 230000 | 230000 | 210000 | 260000 | 280000 | 310000 |
|  | 160000 | 260000 | 260000 | 240000 | 250000 | 260000 | 330000 |
|  | 200000 | 230000 | 210000 | 280000 | 210000 | 290000 | 360000 |
|  | 100000 | 180000 | 220000 | 180000 | 160000 | 260000 | 410000 |
|  | 180000 | 230000 | 230000 | 210000 | 180000 | 220000 | 310000 |
|  | 180000 | 260000 | 260000 | 240000 | 210000 | 280000 | 330000 |

**S1 Table B:** Raw data of *S. aromaticum* essential oil and eugenol action upon biofilm inhibition and against a mature biofilm of *C. albicans* ATCC MYA 2876*.* Data is expressed as CFU/ml of biofilm dry weight (g).

| ***S. aromaticum*** | | | | | | **Eugenol** | | | | |
| --- | --- | --- | --- | --- | --- | --- | --- | --- | --- | --- |
| Biofilm inhibition | MIC | 5x MIC | 10xMIC | Fluconazole | DMSO 1% | MIC | 5x MIC | 10xMIC | Fluconazole | DMSO 1% |
|  | 1.22E+11 | 1.21E+10 | 6.32E+09 | 7.00E+11 | 6.92E+11 | 2.43E+11 | 1.25E+10 | 1.78E+10 | 1.90E+11 | 8.70E+11 |
|  | 4.58E+10 | 5.12E+10 | 5.83E+09 | 4.64E+10 | 2.14E+12 | 1.76E+11 | 2.37E+10 | 1.03E+10 | 2.71E+11 | 1.14E+12 |
|  | 6.15E+10 | 4.07E+10 | 2.73E+09 | 4.00E+10 | 2.20E+12 | 1.89E+11 | 1.81E+10 | 1.60E+10 | 2.62E+11 | 1.02E+12 |
|  | 3.47E+10 | 5.96E+10 | 3.31E+10 | 5.71E+10 | 6.67E+10 | 3.00E+13 | 1.08E+12 | 5.33E+10 | 1.53E+13 | 3.28E+13 |
|  | 3.95E+10 | 8.52E+10 | 2.21E+10 | 2.81E+10 | 5.77E+10 | 2.94E+13 | 1.96E+12 | 3.79E+10 | 1.84E+13 | 2.85E+13 |
|  | 5.00E+10 | 4.13E+10 | 1.00E+10 | 5.88E+10 | 2.50E+10 | 2.92E+13 | 3.37E+12 | 6.00E+10 | 3.54E+13 | 2.38E+13 |
|  | 1.55E+11 | 4.40E+10 | 2.09E+10 | 1.90E+11 | 8.70E+11 | 5.85E+13 | 1.06E+12 | 6.44E+10 | 1.84E+13 | 4.21E+13 |
|  | 2.43E+11 | 2.66E+10 | 1.96E+10 | 2.71E+11 | 1.14E+12 | 1.57E+13 | 1.17E+12 | 7.61E+10 | 7.08E+13 | 5.71E+13 |
|  | 2.37E+11 | 2.51E+10 | 1.39E+10 | 2.62E+11 | 1.02E+12 | 1.92E+13 | 6.59E+11 | 3.57E+10 | 1.74E+13 | 3.06E+13 |
| Mature biofilm | 4.00E+11 | 7.67E+11 | 9.00E+10 | 1.75E+11 | 7.27E+11 | 5.20E+11 | 3.40E+10 | 1E+09 | 1.429E+09 | 1.79E+10 |
|  | 3.25E+11 | 5.18E+10 | 2.60E+10 | 1.75E+11 | 1.36E+12 | 6.20E+11 | 3.00E+10 | 315789474 | 396039604 | 1.18E+11 |
|  | 6.00E+11 | 5.77E+10 | 3.20E+10 | 1.63E+11 | 1.00E+12 | 8.00E+11 | 4.25E+10 | 12269939 | 2.273E+09 | 3.33E+10 |
|  | 5.13E+10 | 2.87E+10 | 4.94E+09 | 1.429E+09 | 1.79E+10 | 1.28E+13 | 1.77E+11 | 7.21E+10 | 9.91E+12 | 6.35E+12 |
|  | 9.10E+11 | 3.38E+10 | 5.19E+09 | 396039604 | 1.18E+11 | 1.78E+13 | 3.16E+11 | 5.20E+10 | 1.73E+13 | 6.26E+12 |
|  | 8.91E+10 | 2.18E+10 | 6.12E+09 | 2.273E+09 | 3.33E+10 | 1.05E+13 | 2.67E+11 | 6.45E+10 | 1.06E+13 | 5.33E+12 |
|  | 6.81E+11 | 1.18E+12 | 8.59E+09 | 9.91E+12 | 6.35E+12 | 6.32E+12 | 6.90E+10 | 5E+09 | 1.17E+12 | 4.10E+12 |
|  | 6.92E+12 | 1.42E+12 | 3.36E+09 | 1.73E+13 | 6.26E+12 | 6.07E+12 | 1.75E+10 | 1.299E+09 | 1.34E+12 | 1.00E+13 |
|  | 6.99E+12 | 5.91E+11 | 3.17E+09 | 1.06E+13 | 5.33E+12 | 1.97E+12 | 1.30E+10 | 970873786 | 2.47E+12 | 2.30E+12 |

**S1 Table C:** Raw data of cytotoxic effect of *S. aromaticum* essential oil on TR146 and THP-1 cells after 24 hours of treatment.

| **THP-1** | **2500 µg/mL** | **250 µg/mL** | **25 µg/mL** | **2.5 µg/mL** | **0.25 µg/mL** | **DMSO 1%** | **Cell** |
| --- | --- | --- | --- | --- | --- | --- | --- |
|  | 20.05669 | 20.75799 | 86.29145 | 91.3533 | 82.13277 | 105.6955 | 99.49565 |
|  | 16.0889 | 20.06971 | 81.23795 | 81.06441 | 79.38862 | 108.9471 | 93.17525 |
|  | 17.52593 | 19.45562 | 45.19486 | 40.74597 | 72.16892 | 116.9426 | 100.7311 |
|  | 15.16681 | 18.44164 | 63.89159 | 63.61598 | 77.06022 | 106.2287 | 106.598 |
|  | 11.93373 | 17.23075 | 70.57978 | 76.40728 | 83.42213 | 86.2094 | 100.0606 |
|  | 12.41823 | 17.65053 | 69.30419 | 74.43928 | 81.1657 | 93.19263 | 97.73307 |
|  | 11.14269 | 17.81759 | 62.19517 | 71.4718 | 79.67282 | 86.06967 | 101.5041 |
|  | 11.14486 | 18.83025 | 65.79619 | 74.84183 | 74.39371 | 85.01536 | 100.7022 |
| **TR146** | 3.474971 | 3.634337 | 88.20601 | 92.7058 | 95.74864 | 99.55555 | 105.47798 |
|  | 3.490557 | 3.636936 | 83.46411 | 90.30192 | 93.47868 | 97.1649 | 97.79396 |
|  | 3.493797 | 3.578173 | 81.71399 | 91.15566 | 93.18876 | 98.23079 | 98.25917 |
|  | 3.493797 | 3.551789 | 80.70146 | 89.97755 | 93.77123 | 105.5155 | 96.22497 |
|  | 3.672577 | 4.363624 | 74.87507 | 78.59302 | 97.01291 | 92.63059 | 107.97109 |
|  | 3.759884 | 5.964952 | 73.8724 | 77.76385 | 100.7649 | 102.5891 | 89.10154 |
|  | 3.782111 | 5.495359 | 74.32919 | 81.5178 | 100.1429 | 103.993 | 90.50221 |
|  | 3.845926 | 6.527376 | 74.34866 | 82.64092 | 101.1685 | 100.7873 | 92.4382 |

**S1 Table D:** Raw data of cytotoxic effect of eugenol on TR146 and THP-1 cells after 24 hours of treatment.

| **THP-1** | **2500 µg/mL** | **250 µg/mL** | **25 µg/mL** | **2.5 µg/mL** | **0.25 µg/mL** | **DMSO 1%** | **Cell** |
| --- | --- | --- | --- | --- | --- | --- | --- |
|  | 20.05669 | 20.75799 | 86.29145 | 91.3533 | 82.13277 | 105.6955 | 99.49565 |
|  | 16.0889 | 20.06971 | 81.23795 | 81.06441 | 79.38862 | 108.9471 | 93.17525 |
|  | 17.52593 | 19.45562 | 45.19486 | 40.74597 | 72.16892 | 116.9426 | 100.7311 |
|  | 15.16681 | 18.44164 | 63.89159 | 63.61598 | 77.06022 | 106.2287 | 106.598 |
|  | 11.93373 | 17.23075 | 70.57978 | 76.40728 | 83.42213 | 86.2094 | 100.0606 |
|  | 12.41823 | 17.65053 | 69.30419 | 74.43928 | 81.1657 | 93.19263 | 97.73307 |
|  | 11.14269 | 17.81759 | 62.19517 | 71.4718 | 79.67282 | 86.06967 | 101.5041 |
|  | 11.14486 | 18.83025 | 65.79619 | 74.84183 | 74.39371 | 85.01536 | 100.7022 |
| **TR146** | 3.939551 | 20.680834 | 80.31805 | 84.30627 | 104.0652 | 99.36429 | 99.36429 |
|  | 4.033205 | 16.398569 | 79.24249 | 83.41682 | 108.0899 | 110.0467 | 110.0467 |
|  | 4.057048 | 15.894839 | 79.73248 | 87.44366 | 107.4227 | 111.5527 | 111.5527 |
|  | 4.125502 | 27.001878 | 79.75337 | 88.64843 | 108.5229 | 108.114 | 108.114 |
|  | 3.939551 | 24.680834 | 80.31805 | 84.30627 | 104.0652 | 99.36429 | 99.36429 |
|  | 4.033205 | 16.398569 | 79.24249 | 83.41682 | 108.0899 | 110.0467 | 110.0467 |
|  | 4.057048 | 15.894839 | 79.73248 | 87.44366 | 107.4227 | 111.5527 | 111.5527 |
|  | 4.125502 | 17.001878 | 79.75337 | 88.64843 | 108.5229 | 108.114 | 108.114 |

**S1 Table E:** Raw data used to plot the Kaplan-meier survival analysis on *S. aromaticum* (MIC, 2xMIC, 5xMIC, 10xMIC, 15xMIC, and 20xMIC) *in vivo* toxicity using *G. mellonella* model.

|  | Time | MIC | 2xMIC | 5xMIC | 10xMIC | 15xMIC | 20xMIC | DMSO  100% | DMSO  1% | Injection  only |
| --- | --- | --- | --- | --- | --- | --- | --- | --- | --- | --- |
| MIC | 96 | 0 |  |  |  |  |  |  |  |  |
|  | 96 | 0 |  |  |  |  |  |  |  |  |
|  | 96 | 0 |  |  |  |  |  |  |  |  |
|  | 96 | 0 |  |  |  |  |  |  |  |  |
|  | 96 | 0 |  |  |  |  |  |  |  |  |
|  | 96 | 0 |  |  |  |  |  |  |  |  |
|  | 96 | 0 |  |  |  |  |  |  |  |  |
|  | 96 | 0 |  |  |  |  |  |  |  |  |
|  | 96 | 0 |  |  |  |  |  |  |  |  |
|  | 96 | 0 |  |  |  |  |  |  |  |  |
| 2xMIC | 96 |  | 0 |  |  |  |  |  |  |  |
|  | 96 |  | 0 |  |  |  |  |  |  |  |
|  | 96 |  | 0 |  |  |  |  |  |  |  |
|  | 96 |  | 0 |  |  |  |  |  |  |  |
|  | 96 |  | 0 |  |  |  |  |  |  |  |
|  | 96 |  | 0 |  |  |  |  |  |  |  |
|  | 96 |  | 0 |  |  |  |  |  |  |  |
|  | 96 |  | 0 |  |  |  |  |  |  |  |
|  | 96 |  | 0 |  |  |  |  |  |  |  |
|  | 96 |  | 0 |  |  |  |  |  |  |  |
| 5xMIC | 96 |  |  | 0 |  |  |  |  |  |  |
|  | 96 |  |  | 0 |  |  |  |  |  |  |
|  | 96 |  |  | 0 |  |  |  |  |  |  |
|  | 96 |  |  | 0 |  |  |  |  |  |  |
|  | 96 |  |  | 0 |  |  |  |  |  |  |
|  | 96 |  |  | 0 |  |  |  |  |  |  |
|  | 96 |  |  | 0 |  |  |  |  |  |  |
|  | 96 |  |  | 0 |  |  |  |  |  |  |
|  | 96 |  |  | 0 |  |  |  |  |  |  |
|  | 96 |  |  | 0 |  |  |  |  |  |  |
| 10xMIC | 96 |  |  |  | 0 |  |  |  |  |  |
|  | 96 |  |  |  | 0 |  |  |  |  |  |
|  | 96 |  |  |  | 0 |  |  |  |  |  |
|  | 96 |  |  |  | 0 |  |  |  |  |  |
|  | 96 |  |  |  | 0 |  |  |  |  |  |
|  | 96 |  |  |  | 0 |  |  |  |  |  |
|  | 96 |  |  |  | 0 |  |  |  |  |  |
|  | 96 |  |  |  | 0 |  |  |  |  |  |
|  | 96 |  |  |  | 0 |  |  |  |  |  |
|  | 96 |  |  |  | 0 |  |  |  |  |  |
| 15xMIC | 96 |  |  |  |  | 0 |  |  |  |  |
|  | 96 |  |  |  |  | 0 |  |  |  |  |
|  | 96 |  |  |  |  | 0 |  |  |  |  |
|  | 96 |  |  |  |  | 0 |  |  |  |  |
|  | 96 |  |  |  |  | 0 |  |  |  |  |
|  | 96 |  |  |  |  | 0 |  |  |  |  |
|  | 96 |  |  |  |  | 0 |  |  |  |  |
|  | 96 |  |  |  |  | 0 |  |  |  |  |
|  | 96 |  |  |  |  | 0 |  |  |  |  |
|  | 96 |  |  |  |  | 0 |  |  |  |  |
| 20xMIC | 96 |  |  |  |  |  | 0 |  |  |  |
|  | 96 |  |  |  |  |  | 0 |  |  |  |
|  | 96 |  |  |  |  |  | 0 |  |  |  |
|  | 96 |  |  |  |  |  | 0 |  |  |  |
|  | 96 |  |  |  |  |  | 0 |  |  |  |
|  | 96 |  |  |  |  |  | 0 |  |  |  |
|  | 96 |  |  |  |  |  | 0 |  |  |  |
|  | 96 |  |  |  |  |  | 0 |  |  |  |
|  | 96 |  |  |  |  |  | 0 |  |  |  |
|  | 96 |  |  |  |  |  | 0 |  |  |  |
| DMSO 100% | 12 |  |  |  |  |  |  | 1 |  |  |
|  | 24 |  |  |  |  |  |  | 1 |  |  |
|  | 24 |  |  |  |  |  |  | 1 |  |  |
|  | 24 |  |  |  |  |  |  | 1 |  |  |
|  | 36 |  |  |  |  |  |  | 1 |  |  |
|  | 36 |  |  |  |  |  |  | 1 |  |  |
|  | 36 |  |  |  |  |  |  | 1 |  |  |
|  | 36 |  |  |  |  |  |  | 1 |  |  |
|  | 48 |  |  |  |  |  |  | 1 |  |  |
|  | 48 |  |  |  |  |  |  | 1 |  |  |
| DMSO  1% | 96 |  |  |  |  |  |  |  | 0 |  |
|  | 96 |  |  |  |  |  |  |  | 0 |  |
|  | 96 |  |  |  |  |  |  |  | 0 |  |
|  | 96 |  |  |  |  |  |  |  | 0 |  |
|  | 96 |  |  |  |  |  |  |  | 0 |  |
|  | 96 |  |  |  |  |  |  |  | 0 |  |
|  | 96 |  |  |  |  |  |  |  | 0 |  |
|  | 96 |  |  |  |  |  |  |  | 0 |  |
|  | 96 |  |  |  |  |  |  |  | 0 |  |
|  | 96 |  |  |  |  |  |  |  | 0 |  |
| Injection only | 96 |  |  |  |  |  |  |  |  | 0 |
|  | 96 |  |  |  |  |  |  |  |  | 0 |
|  | 96 |  |  |  |  |  |  |  |  | 0 |
|  | 96 |  |  |  |  |  |  |  |  | 0 |
|  | 96 |  |  |  |  |  |  |  |  | 0 |
|  | 96 |  |  |  |  |  |  |  |  | 0 |
|  | 96 |  |  |  |  |  |  |  |  | 0 |
|  | 96 |  |  |  |  |  |  |  |  | 0 |
|  | 96 |  |  |  |  |  |  |  |  | 0 |
|  | 96 |  |  |  |  |  |  |  |  | 0 |

**S1 Table F:** *G. mellonella* health index after *S. aromaticum* (MIC, 2xMIC, 5xMIC, 10xMIC, 15xMIC, and 20xMIC) treatment.

| **Time points** | ***G. mellonella* health index** | | | | | | | | |
| --- | --- | --- | --- | --- | --- | --- | --- | --- | --- |
|  | MIC | 2xMIC | 5xMIC | 10xMIC | 15xMIC | 20xMIC | DMSO  100% | DMSO  1% | Injection  only |
| 4h | 10 | 10 | 10 | 10 | 10 | 10 | 9 | 10 | 10 |
|  | 10 | 10 | 10 | 10 | 10 | 10 | 9 | 10 | 10 |
|  | 10 | 10 | 10 | 10 | 10 | 10 | 9 | 10 | 10 |
|  | 10 | 10 | 10 | 10 | 10 | 10 | 10 | 10 | 10 |
|  | 10 | 10 | 10 | 10 | 10 | 10 | 10 | 10 | 10 |
|  | 10 | 10 | 10 | 10 | 10 | 10 | 10 | 10 | 10 |
|  | 10 | 10 | 10 | 10 | 10 | 10 | 10 | 10 | 10 |
|  | 10 | 10 | 10 | 10 | 10 | 10 | 10 | 10 | 10 |
|  | 10 | 10 | 10 | 10 | 10 | 10 | 10 | 10 | 10 |
|  | 10 | 10 | 10 | 10 | 10 | 10 | 10 | 10 | 10 |
| 8h | 10 | 10 | 10 | 10 | 10 | 10 | 9 |  |  |
|  | 10 | 10 | 10 | 10 | 10 | 10 | 9 | 10 | 10 |
|  | 10 | 10 | 10 | 10 | 10 | 10 | 9 | 10 | 10 |
|  | 10 | 10 | 10 | 10 | 10 | 10 | 9 | 10 | 10 |
|  | 10 | 10 | 10 | 10 | 10 | 10 | 9 | 10 | 10 |
|  | 10 | 10 | 10 | 10 | 10 | 10 | 9 | 10 | 10 |
|  | 10 | 10 | 10 | 10 | 10 | 10 | 9 | 10 | 10 |
|  | 10 | 10 | 10 | 10 | 10 | 10 | 8 | 10 | 10 |
|  | 10 | 10 | 10 | 10 | 10 | 10 | 8 | 10 | 10 |
|  | 10 | 10 | 10 | 10 | 10 | 10 | 8 | 10 | 10 |
| 12h | 10 | 10 | 10 | 10 | 10 | 10 | 0 | 9 | 10 |
|  | 10 | 10 | 10 | 10 | 10 | 10 | 5 | 9 | 10 |
|  | 10 | 10 | 10 | 10 | 10 | 10 | 4 | 9 | 10 |
|  | 10 | 10 | 10 | 10 | 10 | 10 | 5 | 10 | 10 |
|  | 10 | 10 | 10 | 10 | 10 | 10 | 5 | 10 | 10 |
|  | 10 | 10 | 10 | 10 | 10 | 10 | 4 | 10 | 10 |
|  | 10 | 10 | 10 | 10 | 10 | 10 | 3 | 10 | 10 |
|  | 10 | 10 | 10 | 10 | 10 | 10 | 5 | 10 | 10 |
|  | 10 | 10 | 10 | 10 | 10 | 10 | 5 | 10 | 10 |
|  | 10 | 10 | 10 | 10 | 10 | 10 | 4 | 10 | 10 |
| 24h | 10 | 9 | 9 | 9 | 9 | 9 | 0 | 9 | 10 |
|  | 10 | 9 | 9 | 9 | 9 | 9 | 3 | 9 | 10 |
|  | 10 | 9 | 9 | 9 | 9 | 9 | 2 | 9 | 10 |
|  | 10 | 10 | 10 | 9 | 9 | 9 | 4 | 10 | 10 |
|  | 10 | 10 | 10 | 10 | 10 | 10 | 4 | 10 | 10 |
|  | 10 | 10 | 10 | 10 | 10 | 10 | 0 | 10 | 10 |
|  | 10 | 10 | 10 | 10 | 10 | 10 | 0 | 10 | 10 |
|  | 10 | 10 | 10 | 10 | 10 | 10 | 3 | 10 | 10 |
|  | 10 | 10 | 10 | 10 | 10 | 10 | 2 | 10 | 10 |
|  | 10 | 10 | 10 | 10 | 10 | 10 | 0 | 10 | 10 |
| 36h | 10 | 9 | 9 | 9 | 9 | 9 | 0 | 9 | 9 |
|  | 10 | 9 | 9 | 9 | 9 | 9 | 0 | 9 | 9 |
|  | 10 | 9 | 9 | 9 | 9 | 9 | 2 | 9 | 10 |
|  | 10 | 10 | 10 | 9 | 9 | 9 | 0 | 9 | 10 |
|  | 10 | 10 | 10 | 10 | 10 | 10 | 2 | 10 | 10 |
|  | 10 | 10 | 10 | 10 | 10 | 10 | 0 | 10 | 10 |
|  | 10 | 10 | 10 | 10 | 10 | 10 | 0 | 10 | 10 |
|  | 10 | 10 | 10 | 10 | 10 | 10 | 0 | 10 | 10 |
|  | 10 | 10 | 10 | 10 | 10 | 10 | 0 | 10 | 10 |
|  | 10 | 10 | 10 | 10 | 10 | 10 | 0 | 10 | 10 |
| 48h | 9 | 9 | 9 | 9 | 9 | 9 | 0 | 9 | 9 |
|  | 9 | 9 | 9 | 9 | 9 | 9 | 0 | 9 | 9 |
|  | 9 | 9 | 9 | 9 | 9 | 9 | 0 | 9 | 10 |
|  | 10 | 9 | 9 | 9 | 9 | 9 | 0 | 9 | 10 |
|  | 10 | 9 | 10 | 10 | 9 | 10 | 0 | 9 | 10 |
|  | 10 | 10 | 10 | 10 | 10 | 10 | 0 | 10 | 10 |
|  | 10 | 10 | 10 | 10 | 10 | 10 | 0 | 10 | 10 |
|  | 10 | 10 | 10 | 10 | 10 | 10 | 0 | 10 | 10 |
|  | 10 | 10 | 10 | 10 | 10 | 10 | 0 | 10 | 10 |
|  | 10 | 10 | 10 | 10 | 10 | 10 | 0 | 10 | 10 |
| 72h | 9 | 9 | 9 | 9 | 9 | 9 | 0 | 9 | 9 |
|  | 9 | 9 | 9 | 9 | 9 | 9 | 0 | 9 | 9 |
|  | 9 | 9 | 9 | 9 | 9 | 9 | 0 | 9 | 10 |
|  | 10 | 9 | 9 | 9 | 9 | 9 | 0 | 9 | 10 |
|  | 10 | 9 | 10 | 10 | 9 | 10 | 0 | 9 | 10 |
|  | 9 | 9 | 9 | 9 | 9 | 9 | 0 | 9 | 9 |
|  | 9 | 9 | 9 | 9 | 9 | 9 | 0 | 9 | 9 |
|  | 9 | 9 | 9 | 9 | 9 | 9 | 0 | 9 | 10 |
|  | 10 | 9 | 9 | 9 | 9 | 9 | 0 | 9 | 10 |
|  | 10 | 10 | 10 | 10 | 10 | 10 | 0 | 10 | 10 |
| 96 | 9 | 9 | 9 | 9 | 9 | 9 | 0 | 9 | 9 |
|  | 9 | 9 | 9 | 9 | 9 | 9 | 0 | 9 | 9 |
|  | 9 | 9 | 9 | 9 | 9 | 9 | 0 | 9 | 9 |
|  | 9 | 9 | 9 | 9 | 9 | 9 | 0 | 9 | 9 |
|  | 9 | 9 | 9 | 9 | 9 | 9 | 0 | 9 | 9 |
|  | 9 | 9 | 9 | 9 | 9 | 9 | 0 | 9 | 9 |
|  | 9 | 9 | 9 | 9 | 9 | 9 | 0 | 9 | 9 |
|  | 9 | 9 | 9 | 9 | 9 | 9 | 0 | 9 | 10 |
|  | 10 | 9 | 9 | 9 | 9 | 9 | 0 | 9 | 10 |
|  | 10 | 10 | 10 | 10 | 10 | 10 | 0 | 10 | 10 |
